# Multivariate autoregressive models with exogenous inputs for intracerebral responses to direct electrical stimulation of the human brain

**DOI:** 10.3389/fnhum.2012.00317

**Published:** 2012-11-30

**Authors:** Jui-Yang Chang, Andrea Pigorini, Marcello Massimini, Giulio Tononi, Lino Nobili, Barry D. Van Veen

**Affiliations:** ^1^Department of Electrical and Computer Engineering, University of WisconsinMadison, WI, USA; ^2^Department of Clinical Sciences, University of MilanMilan, Italy; ^3^Department of Psychiatry, University of WisconsinMadison, WI, USA; ^4^Centre of Epilepsy Surgery “C. Munari”, Niguarda HospitalMilan, Italy

**Keywords:** intracerebal EEG, evoked response, MVARX model, cross-validation, integrated information

## Abstract

A multivariate autoregressive (MVAR) model with exogenous inputs (MVARX) is developed for describing the cortical interactions excited by direct electrical current stimulation of the cortex. Current stimulation is challenging to model because it excites neurons in multiple locations both near and distant to the stimulation site. The approach presented here models these effects using an exogenous input that is passed through a bank of filters, one for each channel. The filtered input and a random input excite a MVAR system describing the interactions between cortical activity at the recording sites. The exogenous input filter coefficients, the autoregressive coefficients, and random input characteristics are estimated from the measured activity due to current stimulation. The effectiveness of the approach is demonstrated using intracranial recordings from three surgical epilepsy patients. We evaluate models for wakefulness and NREM sleep in these patients with two stimulation levels in one patient and two stimulation sites in another resulting in a total of 10 datasets. Excellent agreement between measured and model-predicted evoked responses is obtained across all datasets. Furthermore, one-step prediction is used to show that the model also describes dynamics in pre-stimulus and evoked recordings. We also compare integrated information—a measure of intracortical communication thought to reflect the capacity for consciousness—associated with the network model in wakefulness and sleep. As predicted, higher information integration is found in wakefulness than in sleep for all five cases.

## 1. Introduction

The remarkable cognitive abilities of the healthy human brain depend on an exquisite balance between functional specialization of local cortical circuits and their functional integration through long-range connections. Hence, there is considerable interest in characterizing long-range cause and effect or directional interactions in the human brain. Multivariate autoregressive (MVAR) models, sometimes referred to as vector autoregressive (VAR) models, have been widely applied to study directional cortical network properties from both intracranial data (e.g., Bernasconi and König, 1999; Brovelli et al., 2004; Winterhalder et al., 2005; Ding et al., 2006; Korzeniewska et al., 2008) and scalp EEG or MEG (e.g., Babiloni et al., 2005; Malekpour et al., 2012). An MVAR model describes each signal as a weighted combination of its own past values and the past values of other signals in the model—an autoregression—plus an error term. The weights relating the present of one signal to the past of another capture the causal or directed influence between signals. A variety of different metrics for summarizing the directed interactions in MVAR models have been proposed, including directed transfer functions (Kamiński and Blinowska, 1991), directed coherence (Baccalá and Sameshima, 2001), conditional Granger causality (Geweke, 1984), and integrated information (Barrett and Seth, 2011).

MVAR models assume the data is stationary and of constant mean. While stationarity and constant mean may be reasonable assumptions for a relatively short duration of spontaneous data, evoked or event-related data appear to violate these assumptions. For example, the mean or average response to a stimulus varies with time. An MVAR model fit to data with a time-varying mean results in spurious interactions because the assumption of stationarity is violated. Adaptive or time-varying methods have been developed to relax stationarity assumptions (Ding et al., 2000; Möller et al., 2001; Astolfi et al., 2008). For example, a time-varying mean response is removed by subtracting the ensemble average (Ding et al., 2000) and the MVAR model parameters are allowed to vary with time. Adaptive models require specification of an adaptation rate parameter that effectively determines how much of the past data is used to estimate the present model parameters, or equivalently, how fast the model is changing. Models that use fast adaptation are able to track faster changes in the underlying data, but employ less data to estimate model parameters and consequently possess more variability in the estimated model parameters (see (Astolfi et al., 2008), for assessment of these issues).

During the pre-surgical evaluation of drug-resistant epileptic patients, direct electrical stimulation of the brain is systematically performed for diagnostic purposes to identify the epileptogenic zone (Munari et al., 1994). Electrical stimulation generates a time-varying response at the recording sites. In this paper we propose describing the response of the brain using stationary MVAR models with an exogenous input (MVARX) derived from the stimulus characteristics. MVARX models are commonly used in econometric time series analysis (Lütkepohl, 2006). The advantage of the MVARX model is that it does not require subtraction of the mean and consequent reduction in signal-to-noise ratio (SNR) or the complication of time-varying models to capture the response evoked by direct electrical stimulation. The model captures both the mean evoked response and the background activity present during the recordings. We demonstrate the effectiveness of the MVARX model using intracerebral recordings from epilepsy patients.

Direct electrical stimulation of the brain presents several modeling challenges. Although the timing and location of the stimulus is known precisely, the response of the brain in the near vicinity of the stimulus cannot be measured due to electrical artifacts and the propagation of the stimulus to more distant sites depends on the topology of axons in the vicinity of the stimulation site (Ranck, 1975). Electrical stimulation creates action potentials in neurons whose axons pass near the stimulus site. These neurons synapse both near and distant to the stimulation site, so the stimulus actually activates multiple, *a priori* unknown areas. The MVARX model explicitly accounts for this effect with a bank of finite impulse response (FIR) filters that capture the impact of the exogenous input, i.e., stimulus, on all recording sites. The exogenous input filter coefficients and the MVAR model parameters are simultaneously estimated from the recordings and knowledge of the stimulation times using a least squares procedure. The exogenous input filter coefficients describe the conduction paths from the stimulus site to each recording site, while the MVAR model parameters capture the causal interactions between recording sites.

The MVARX model is applied to 10 datasets collected from three subjects in wakefulness and NREM sleep. Two stimulation levels are studied in one subject, and two stimulation sites in another. The data consists of the intracranial response to 30 current impulses separated by 1 s. A cross-validation (CV) procedure is introduced for choosing the memory in the MVARX model. We demonstrate that a stationary MVARX model accurately describes the activity evoked by direct electrical stimulation. Comparison to a series of univariate autoregressive models with exogenous inputs (ARX) reveals that causal interactions must be modeled to accurately describe the measured activity. The series of ARX models result in much larger modeling error than the MVARX model. One-step prediction performance is used to demonstrate that the MVARX model also captures spontaneous fluctuations in the recorded data. The MVARX model errors pass a whiteness test while the univariate ARX models do not, further supporting the applicability of the MVARX model.

The MVARX models are employed to contrast integrated information in wakefulness and sleep. Integrated information is a measure of the extent to which the information generated by the causal interactions in the model cannot be partitioned into independent subparts of the system. Hence, integrated information measures the balance between functional specialization and integration represented by the model. Theoretical considerations (Tononi, 2004; Laureys, 2005; Dehaene et al., 2006; Seth et al., 2008) indicate that integrated information should be less in sleep than in wakefulness. This prediction is confirmed in all 10 datasets using our MVARX model.

This paper is organized as follows. Section 2 describes the data and preprocessing procedures. Section 3 defines the MVARX model, introduces the method for estimating the model parameters, including our CV approach for selecting model memory, and presents the residual whiteness test. Section 4 demonstrates the effectiveness of the proposed model using the 10 datasets described above and section 5 applies the MVARX models to contrast integrated information in wakefulness and sleep. This paper concludes with a discussion in section 6. For notation, boldface lower and upper case symbols represent vectors and matrices, respectively, while superscript *T* denotes matrix transpose and superscript −1 denotes matrix inverse. The trace of a matrix **A** is tr[**A**] and the determinant is det(**A**). *E*{*a*} denotes the expectation of a random variable *a*. The Euclidean norm of a vector **x** is $||x|{|}_{2}=\sqrt{x^{T}x}$. The number of elements in a set *S* is |*S*|. x~N(μ, Σ) means that the vector **x** is normally distributed with mean μ and covariance matrix Σ.

## 2. Data

### 2.1. Subjects and experimental protocol

Three subjects with long-standing drug-resistant focal epilepsy participated in this study. All patients were candidates for surgical removal of the epileptic focus. During pre-surgical evaluation the patients underwent individual investigation with stereotactically implanted intracerebral multilead electrodes for precise localization of the epileptogenic areas (Cossu et al., 2005). All patients gave written informed consent before intracerebral electrode implantation as approved by the local Ethical Committee. Confirmation of the hypothesized seizure focus and localization of epileptogenic tissue in relation to essential cortex was achieved by simultaneous scalp and intracerebral electrode recording, as well as intracerebral stimulation during wakefulness and sleep to further investigate connectivity of epileptogenic and healthy tissue (Valentín et al., 2002, 2005). The decision on implantation site, duration of implantation and stimulation site(s) was made entirely on clinical needs. Stereoelectroencephalography (SEEG) activity was recorded from platinumiridium semiflexible multilead intracerebral electrodes, with a diameter of 0.8 mm, a contact length of 2 mm, an intercontact distance of 1.5 mm and a maximal contact number of 18 (Dixi Medical, Besançon, France) (Cossu et al., 2005). The individual placement of electrodes was ascertained by post-implantation tomographic imaging (CT) scans. Scalp EEG activity was recorded from two platinum needle electrodes placed during surgery at “10–20” positions Fz and Cz on the scalp. Electroocular activity was registered at the outer canthi of both eyes, and submental electromyographic activity was acquired with electrodes attached to the chin. EEG and SEEG signals were recorded using a 192-channel recording system (Nihon-Kohden Neurofax-110) with a sampling rate of 1000 Hz. Data was recorded and exported in EEG Nihon-Kohden format (Nobili et al., 2011, 2012). The data for each channel is obtained using bipolar referencing to a neighboring contact located entirely in the white matter. Intracerebral stimulations were started on the third day after electrode implantation. In eight out of ten cases we discuss, stimulation of strength 5 mA were performed, while for the other two cases stimulation of 1 mA were applied. At each stimulation session, the stimulation is applied at a single channel and SEEG recordings were obtained from all other channels. A single stimulation session consisted of a 30 impulse stimulation train at intervals of 1 s. Each impulse is of 0.2-ms duration. The channels that were stimulated were chosen based on clinical requirements. All patients included in this study were stimulated during wakefulness and stage 4 of NREM sleep. Sleep staging was performed using standard criteria (Rechtschaffen and Kales, 1968). Stimulations which elicited muscle twitches, sensations or cognitive symptoms, were excluded from this study, in order to prevent possible awareness of stimulation or alteration of sleep depth.

**Figure 1:**
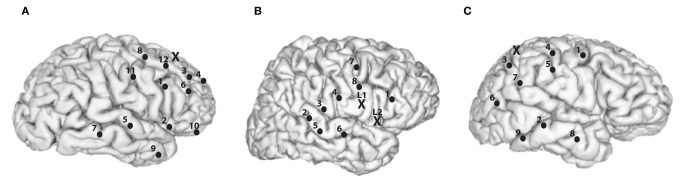
**Recording and stimulation electrode placements for the subjects**. Black dots represents recording channels while black “X” represents stimulating channel(s). **(A)** Subject A, right hemisphere is shown. 1, Inferior frontal opercular; 2, anterior horizontal lateral fissure; 3, middle frontal gyrus; 4, middle frontal sulcus; 5, superior temporal sulcus; 6, inferior frontal sulcus; 7, middle temporal gyrus; 8, middle frontal gyrus; 9, middle temporal gyrus; 10, orbital gyrus; 11, precentral gyrus; 12, superior frontal sulcus; and X, middle frontal gyrus. **(B)** Subject B, right hemisphere is shown. 1, Inferior frontal gyrus; 2, superior temporal sulcus; 3, posterior lateral fissure; 4, postcentral solcus; 5, superior temporal gyrus; 6, transversal temporal sulcus; 7, superior frontal gyrus; 8, subcentral gyrus; X (L1), precentral gyrus, and X (L2), subcentral sulcus. **(C)** Subject C, right hemisphere is shown. 1, Precentral gyrus; 2, posterior middle temporal gyrus; 3, inferior parietal lobule; 4, postcentral gyrus; 5, postcentral sulcus; 6, angular gyrus; 7, supramarginal gyrus; 8, anterior middle temporal gyrus; 9, inferior temporal gyrus, and X, superior parietal lobule.

In our analysis, we consider a subset of 8–12 recording channels of all channels for each subject, as illustrated in Figure 1. The 8–12 channels were selected based on approximately maximizing the distance between the subset of channels that are both artifact free and near the surface of the cortex.

### 2.2. Preprocessing

**Figure 2:**
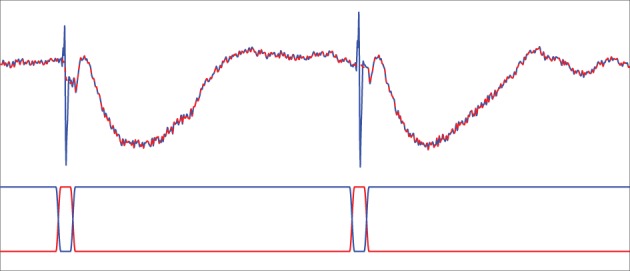
**Tukey-windowed median filtering for eliminating volume conduction artifacts**. The upper trace depicts an example of raw data (blue solid line) and the Tukey-windowed median filter output (red dashed line). The lower trace depicts the weighting applied to the raw data (blue solid line) and the median filtered data (red solid line) to eliminate the volume conduction artifact.

During each stimulation session, a raw trigger signal that indicates the occurrence of current stimulation with 1 and the absence of stimulation with 0 is collected at a sampling rate of 1000 Hz in addition to the SEEG recordings. We use a Tukey-windowed median filter to remove volume conduction artifacts within 39 ms of each stimulus. First, a median filter of order 19 is applied to the raw data channel by channel. Next, the raw data within a 39-ms window centered at each stimulus is replaced with a weighted average of the raw data and the median filtered data to eliminate the artifact. The weights for the median filtered data take the form of a Tukey window (Bloomfield, 2000, p.69) and are zero for ±20 ms away from the stimulus, a cosine rising from 0 to 1 beginning at 19 ms prior to the stimulus and ending at 10 ms prior to the stimulus, unity until 10 ms post-stimulus, and then a cosine decreasing from 1 to 0 ending at 19 ms post-stimulus. The weighting applied to the raw data are one minus those applied to the median filtered data. Figure 2 illustrates the results of this process. The cleaned data is then lowpass filtered by an FIR filter with passband-edge of 48 Hz and stopband-edge of 49.9 Hz to eliminate 50 Hz powerline contamination, and the lowpass filtered data is downsampled by a factor of 10 to a sampling frequency of 100 Hz. The portion of the downsampled data containing responses to stimulation are further segmented into 30 epochs of data **y**^(*j*)^_*n*_, each of which contains 100 samples. Here superscript (*j*) denotes epoch index while subscript *n* denotes time index. The start of each epoch is from 12 samples (0.12 s) before the occurrence of a stimulus and the end is 87 samples (0.87 s) post-stimulus. Similarly, the raw trigger signal is lowpass filtered, downsampled by 10, and partitioned into 100-sample epochs *x*^(*j*)^_*n*_.

In principle, filtering the signal may have an impact on model estimation and causality inference (Barnett and Seth, 2011). We minimize the potential impact of filtering by specifying the stop-band edge of the lowpass filter close to the post-downsampling Nyquist frequency.

### 2.3. Identification of outlying epochs

An automated procedure is employed to exclude epochs that markedly deviate from the majority of epochs due to non-stationary brain activity or other factors. Let **y**^(*j*)^_*n*_ = [*y*^(*j*)^_1, *n*_, *y*^(*j*)^_2, *n*_, …, *y*^(*j*)^_*d*, *n*_]^*T*^ represent the *d* channels of recordings at time *n* = 1, 2, …, *N*_*j*_ from epochs *j* = 1, 2, …, *J*. For epoch *m*, we compute the time-varying mean μ^−*m*^_**y**_(n) and time-varying covariance matrix Σ^−*m*^_**y**_(n) by excluding the *m*-th epoch of data. That is,

(1) $$\mu_{\text{y}}^{-m}(n)=\frac{1}{J-1}\sum_{j\;=\;1,\;j\;\neq \;m}^{J}{\text{y}}_{n}^{\left(j\right)}$$

(2) $$\Sigma_{\text{y}}^{-m}(n)=\frac{1}{J-2}\times \sum_{j\;=\;1,\;j\;\neq \;m}^{J}\text{​}\left({\text{y}}_{n}^{\left(j\right)}-\mu_{\text{y}}^{-m}(n)\right){\left({\text{y}}_{n}^{\left(j\right)}\text{​}-\text{​}\mu_{\text{y}}^{-m}(n)\right)}^{T}\text{​},$$

for *n* = 1, …, 100. Here *m* = 1 to *J* and *J* is 30 for all data sets considered. Then the squared Mahalanobis distance (Penny, 1996) between the epoch *m* and the other epochs is computed as

(3) $$D^{2}(m)=\sum_{n=1}^{100}{\left({\text{y}}_{n}^{\left(m\right)}-\mu_{\text{y}}^{-m}(n)\right)}^{T}\times {\left(\Sigma_{\text{y}}^{-m}(n)\right)}^{-1}\left({\text{y}}_{n}^{\left(m\right)}-\mu_{\text{y}}^{-m}(n)\right).$$

Epochs with *D*^2^(*m*) exceeding

(4) $$100\;\cdot \;d+60\sqrt{2\;\cdot \;100\;\cdot \;d}$$

are declared as outliers and removed from subsequent analysis. Intuitively, if the data is Gaussian, then *D*^2^(*m*) is Chi-squared distributed with 100 · *d* degrees of freedom. This implies that the threshold rules out an epoch *m* if *D*^2^(*m*) exceeds its mean plus 60 standard deviations. Thus this threshold only excludes epochs that have a large deviation from the temporal average of the other epochs. The number of epochs retained for analysis are given in Table 1.

**Table 1:** **Number of non-outlying epochs used in analysis**.

**Dataset**	**Wakefulness epochs**	**Sleep epochs**
Subject A, 1 mA	29	25
Subject A, 5 mA	28	22
Subject B, L1	30	24
Subject B, L2	30	29
Subject C	30	29

## 3. Methods

### 3.1. MVARX model

**Figure 3:**
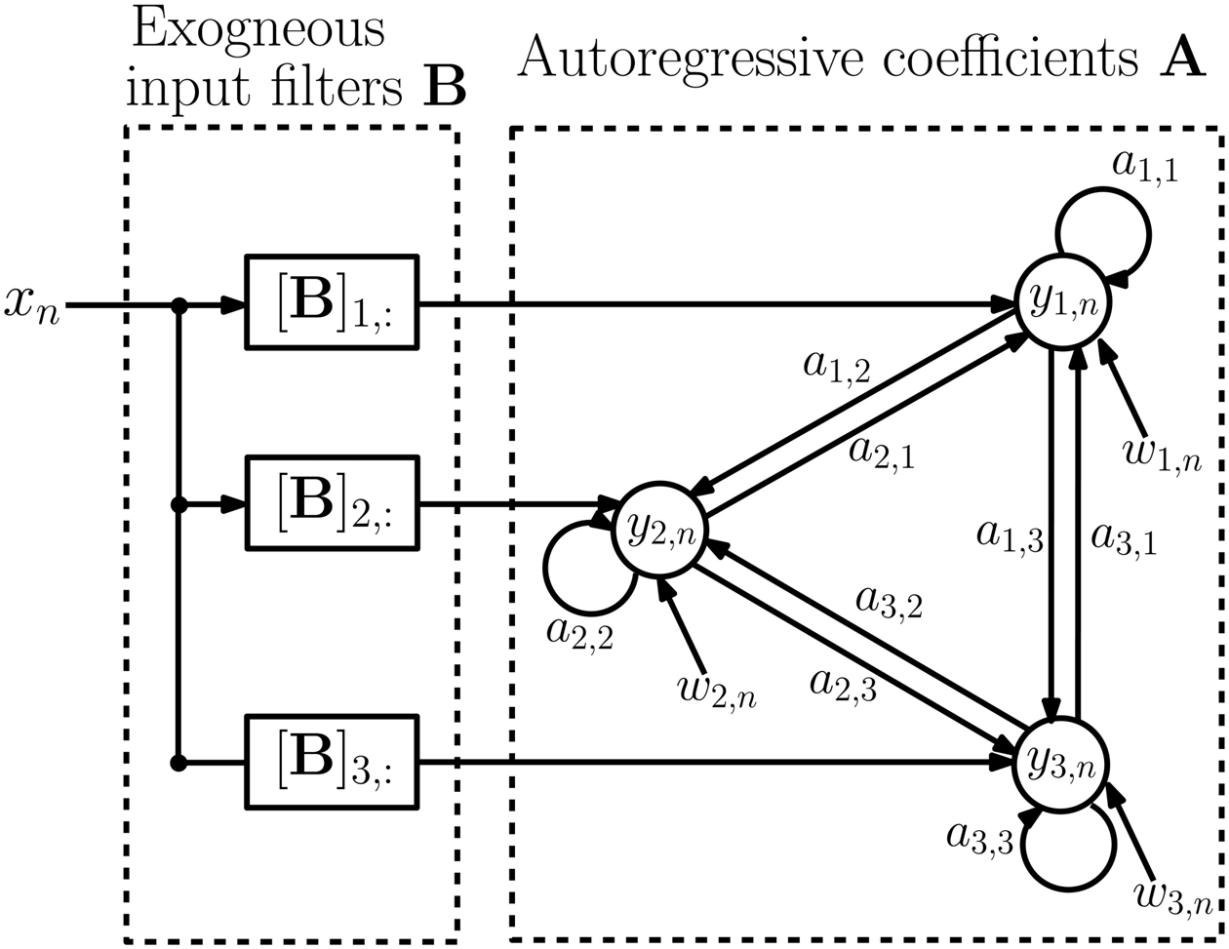
**Schematic diagram of the MVARX model**. *y*_*i, n*_ denotes the recorded signals at the electrodes while *x*_*n*_ represents current stimulation and *w*_*i, n*_ is model error, or equivalently, a random input that generates spontaneous activity. *a*_*i, j*_ captures the *a priori* unknown connectivity between recording sites while [**B**]_*i*,:_ represents the *a priori* unknown transmission characteristics between the stimulus and recording sites.

The MVARX model of order (*p*, ℓ) describes the data as follows (Lütkepohl, 2006):

(5) $${\text{y}}_{n}^{\left(j\right)}=\sum_{i=1}^{p}{\text{ A}}_{i}{\text{y}}_{n-i}^{\left(j\right)}+\sum_{i=0}^{\ell }{\text{ b}}_{i}x_{n-i}^{\left(j\right)}+{\text{ w}}_{n}^{\left(j\right)},$$

where *x*^(*j*)^_*n*_ denotes the input at time *n* and epoch *j*. The *d* × *d* matrices **A**_*i*_ = {*a*_*m*, *n*_(*i*)} contain autoregressive coefficients describing the influence of channel *n* on channel *m* at lag *i*, and the *d* × 1 vectors **b**_*i*_ = {*b*_*m*_(*i*)} contain filter coefficients from the stimulus to channel *m* at lag *i*. The vectors **w**^(*j*)^_*n*_ are *d* × 1 zero-mean noise vectors with covariance matrix **Q** and are assumed to satisfy *E*{**w**^(*i*)^_*n*_(**w**^(*j*)^_s_)^*T*^} = 0, for either *i* ≠ *j* or *n* ≠ *s*. We assume that the epochs are of varying lengths *N*_*j*_ and are possibly disconnected in time to accommodate rejection of outlying epochs. Figure 3 depicts a schematic diagram of an example MVARX model. The diagram assumes there are three recording electrodes corresponding to the recordings *y*_1, *n*_, *y*_2, *n*_, and *y*_3, *n*_ (the epoch index *j* is omitted in the figure for simplicity). The intracranial EEG signals recorded at the electrodes contain contributions due to the current stimulus response and background brain activity. The exogenous input *x*_*n*_ represents the current stimulation. If **B** = [**b**_0_, …, **b**_ℓ_] is a *d* × (ℓ+1) matrix of exogenous input coefficients, then the *i*-th row of **B**, [**B**]_*i*,:_, is the impulse response of the filter representing the unknown transmission characteristics between the current stimulus and the *i*-th recording channel. The autoregressive coefficients **A** = [**A**_1_, …, **A**_*p*_] indicate how past values of the recorded signals affect present values. The autoregressive order *p* determines the time extent of the past that affect the present values and may be regarded as the memory of the system. The signals *w*_1, *n*_, *w*_2, *n*_, and *w*_3, *n*_ can be interpreted as modeling errors or alternatively as a process that generates spontaneous activity.

Electrodes can be used either as stimulating or recording electrodes but cannot be used simultaneously for recording and stimulation. Moreover, the electrodes closest to the stimulation site are affected by huge electrical artifacts and they cannot be used because of consequent low SNR. Hence the recorded data *y*^(*j*)^_*n*_ contains recordings of the effect of the stimulation at distant sites, not the stimulation itself. Stimulation depolarizes the membranes of neurons passing through the neighborhood of the stimulating electrode, possibly creating action potentials in neurons that synapse near the stimulation site and at distant locations (Ranck, 1975), a phenomenon termed fibers of passage. Thus, stimulation generates an “input” that is conveyed to potentially all recording sites in a manner that depends on the axonal topology in the vicinity of the stimulation site. This topology and consequent stimulation effects are usually unknown and described in our MVARX model by the exogenous input filters **B**. In our model we assume the exogenous input is given by the trigger signal associated with delivery of a current pulse, so **B** captures both the shape of the delivered stimulus and the unknown direct propagation of the input to each recording site.

Denote *y*^(*j*)^_*n*, *s*_ and *y*^(*j*)^_*n*, *e*_ as the spontaneous activity and stimulus response to the exogenous input, respectively, at time *n* from epoch *j*. Equation (5) can be alternatively expressed as

(6) $${\text{y}}_{n}^{\left(j\right)}={\text{y}}_{n,\;s}^{\left(j\right)}+{\text{y}}_{n,\;e}^{\left(j\right)}$$

(7) $${\text{y}}_{n,\;s}^{\left(j\right)}=\sum_{i=1}^{p}{\text{A}}_{i}{\text{y}}_{n-i,\;s}^{\left(j\right)}+{\text{ w}}_{n}^{\left(j\right)}$$

(8) $${\text{y}}_{n,\;e}^{\left(j\right)}=\sum_{i=1}^{p}{\text{A}}_{i}{\text{y}}_{n-i,\;e}^{\left(j\right)}+\sum_{i=0}^{\ell }{\text{b}}_{i}x_{n-i}^{\left(j\right)}.$$

Note that in practice *y*^(*j*)^_*n*, *s*_ and *y*^(*j*)^_*n*, *e*_ are not directly observed and cannot be separated from *y*^(*j*)^_*n*_ without knowledge of the MVARX model parameters. The stimulus response component **y**^(*j*)^_*n*, *e*_ is a deterministic term that depends entirely on the stimulus and the model. Given the model parameters Θ = [**A**, **B**], we can generate **y**^(*j*)^_*n*, *e*_ by applying the stimulus sequence **x**^(*j*)^_*n*_ to Equation (8) with zero initial conditions. Recall that **w**^(*j*)^_*n*_ is assumed to be zero mean, so **y**^(*j*)^_*n*, *s*_ is a zero mean random process reflecting the spontaneous component of the recordings. It is common in MVAR modeling to subtract the mean prior to estimating MVAR model parameters (Ding et al., 2000). This corresponds to removing the stimulus response **y**^(*j*)^_*n*, *e*_ and is unnecessary with the MVARX model. We shall assume that the stimulus is repeated multiple times such that averaging **y**^(*j*)^_*n*, *e*_ with respect to the stimulus onset times produces the evoked response of the system. This is not required by the model in Equation (5) but is consistent with conventional electrophysiology practice.

The autoregressive parameters **A** model the inherent neural connectivity between sites—how activity at one site propagates to another site. This is evident in Equations (5–8) by the fact that the **A**_*i*_ are applied to **y**^(*j*)^_*n* − *i*_. If the spontaneous activity **y**^(*j*)^_*n*, *s*_ is very weak relative to **y**^(*j*)^_*n*, *e*_ then the response is described entirely by Equation (8) and the measured data **y**^(*j*)^_*n*_ ≈ y^(*j*)^_*n*, *e*_. In this case there is a potential modeling ambiguity as there are many different combinations of **A**_*i*_ and **b**_*i*_ that could be used to describe **y**^(*j*)^_*n*, *e*_ over a finite duration. For example, **y**^(*j*)^_*n*, *e*_ can be described on 1 ≤ *n* ≤ ℓ + 1 by setting **A**_*i*_ = 0 and only using **b**_*i*_. We control potential ambiguities associated with relatively weak spontaneous activity by limiting ℓ to a value commensurate with the expected duration of stimulus propagation through fibers of passage. This ensures that **b** is not able to capture long duration interactions associated with feed forward and feedback connectivity between sites. Based on previous experimental evidence (Matsumoto et al., 2004), we set ℓ = 10 to accommodate a 100 ms duration of propagation through fibers of passage. We will discuss this choice more thoroughly in section 6.

### 3.2. Estimation of MVARX model parameters

Suppose that we have the recordings and inputs {(**y**^(*j*)^_*n*_, *x*^(*j*)^_*n*_): *j* = 1, 2, …, *J*, *n* = 1, 2, …, *N*_*j*_} for *J* epochs of *N*_*j*_ samples each. Denote *n*_0_ = max(*p*, ℓ), and suppose that *N*_*j*_ ≥ *n*_0_ + 1, for all *j*. Using the first *n*_0_ samples as the initial values, the model in Equation (5) can be rewritten in a simplified form:

(9) $${\text{y}}_{n}^{\left(j\right)}=\Theta {\text{ z}}_{n-1}^{\left(j\right)}+{\text{w}}_{n}^{\left(j\right)},$$

for *j* = 1, …, *J*, *n* = *n*_0_+1, …, *N*_*j*_, where the *d* × (*dp* + ℓ + 1) matrix Θ = [**A**, **B**] and the vector of dimension *dp*+ℓ + 1, **z**^(*j*)^_*n* − 1_ = [(**y**^(*j*)^_*n* − 1_)^*T*^, (**y**^(*j*)^_*n* − 2_)^*T*^, …, (**y**^(*j*)^_*n* − *p*_)^*T*^, *x*^(*j*)^_*n*_, *x*^(*j*)^_*n* − 1_, …, *x*^(*j*)^_*n* − ℓ_]^*T*^. The vectors **y**^(*j*)^_*n*_, **w**^(*j*)^_*n*_, and **z**^(*j*)^_*n* − 1_ can be further concatenated as columns of the matrices **Y**_*j*_, **Z**_*j*_, and **W**_*j*_ to write:

(10) $${\text{Y}}_{j}=\Theta {\text{Z}}_{j}+{\text{W}}_{j}$$

where **Y**_*j*_ = [**y**^(*j*)^_*n*_0_ + 1_, …, **y**^(*j*)^_*N*_*j*__], **Z**_*j*_ = [**z**^(*j*)^_*n*_0__, …, **z**^(*j*)^_*N*_*j*_ − 1_], and **W**_*j*_ = [**w**^(*j*)^_*n*_0_ + 1_, …, **w**^(*j*)^_*N*_*j*__]. This expression takes the form of a linear regression model, and we can obtain an ordinary least square (OLS) estimate of (Θ, **Q**) as (Lütkepohl, 2006, chap. 10.3):

(11) $$\begin{array}{l} \hat{\Theta }=\left(\sum_{j=1}^{J}{\text{Y}}_{j}{\text{Z}}_{j}^{T}\right){\left(\sum_{j=1}^{J}{\text{Z}}_{j}{\text{Z}}_{j}^{T}\right)}^{-1},\\ \hat{\text{Q}}=\frac{1}{N_{t}}\sum_{j=1}^{J}\left({\text{Y}}_{j}-\hat{\Theta }{\text{Z}}_{j}\right){\left({\text{Y}}_{j}-\hat{\Theta }{\text{Z}}_{j}\right)}^{T}, \end{array}$$

where *N*_*t*_ = ∑^*J*^_*j* = 1_ N_*j*_ − *n*_0_*J*. If **w**^(*j*)^_*n*_ is Gaussian, then the OLS estimate $\left(\hat{\Theta },\hat{\text{Q}}\right)$ is also the maximum-likelihood estimate of (Θ,**Q**) (Lütkepohl, 2006).

### 3.3. Model selection with cross-validation

In practice the order *p* could be chosen using numerous different model selection criteria, including Akaike information criterion and the Bayesian information criterion (McQuarrie and Tsai, 1998; Lütkepohl, 2006). Here we use CV to determine *p* in a data-driven fashion [see Cheung et al. (2012) for another example of using CV to select model parameters with neurophysiological data]. The data **y**^(*j*)^_*n*_ and input *x*^(*j*)^_*n*_ are partitioned into training and test sets. The goal is to choose the value *p* that produces the best prediction of test data when the model Θ = [**A**, **B**] is estimated from the training data. We consider two components in assessing model predictive capability. The first is the one-step prediction error, a measure of the model's ability to track the sample-to-sample and epoch-to-epoch fluctuations in the data. The second is the error between the average evoked response predicted by the model and the measured average response. This measures the quality of the model's response to the stimulus.

Partition the epochs of available data into training sets *R*_*m*_ and test sets *S*_*m*_ and assume there are *m* = 1, 2, …, M such partitions. Assume the sets *S*_*m*_ are non-overlapping and are of approximately the same size. Let Θ_*m*_ be the model estimated from *R*_*m*_ as described in the preceding subsection. The one-step prediction error at time *n*, **e**^(*j*)^_*n*_(Θ_*m*_) is the difference between the recording **y**^(*j*)^_*n*_ and the one-step prediction made by Θ_*m*_ using the *n*_0_ samples prior to time *n*, that is, **z**^(*j*)^_*n* − 1_:

(12) $${\text{e}}_{n}^{\left(j\right)}(\Theta_{m})={\text{y}}_{n}^{\left(j\right)}-{\hat{\text{y}}}_{n}^{\left(j\right)}(\Theta_{m})$$

where the one-step prediction ${\hat{\text{y}}}_{n}^{\left(j\right)}(\Theta_{m})=\Theta_{m}{\text{z}}_{n-1}^{\left(j\right)}$. Similarly we define the average response error as

(13) $$\epsilon_{n}(\Theta_{m})={\bar{\text{y}}}_{n}(S_{m})-{\hat{\bar{\text{y}}}}_{n}(\Theta_{m},\;S_{m})$$

where the average evoked response ${\bar{\text{y}}}_{n}(S_{m})=1/|S_{m}|\cdot \sum_{j\in S_{m}}{\text{y}}_{n}^{\left(j\right)}$ and the average model response ${\hat{\bar{\text{y}}}}_{n}(\Theta_{m},\;S_{m})$ over epochs in *S*_*m*_, ${\hat{\bar{\text{y}}}}_{n}(\Theta_{m},\;S_{m})=1/|S_{m}|\cdot \sum_{j\in S_{m}}{\text{y}}_{n,\;e}^{\left(j\right)}(\Theta_{m})$. Here **y**^(*j*)^_*n*, *e*_(Θ_*m*_) is generated using Θ_*m*_ as described following Equation (8). We define a CV score as a weighted combination of the one-step prediction and average response errors averaged over all training/test data partitions

(14) $$\text{CV}(p)=\frac{1}{M}\sum_{m=1}^{M}\left[\frac{{\text{CV}}_{e}(p,\;m)}{w_{e}}+\frac{{\text{CV}}_{\epsilon }(p,\;m)}{w_{\epsilon }}\right]$$

where CV_*e*_(*p*, *m*) is the mean square one-step prediction error of a *p*-th order model Θ_*m*_(*p*) in predicting data in *S*_*m*_:

(15) $${\text{CV}}_{e}(p,\;m)=\frac{1}{\left|S_{m}\right|}\sum_{j\in S_{m}}\frac{1}{N_{j}-n_{0}}\sum_{n=n_{0}+1}^{N_{j}}{\left\|{\text{e}}_{n}^{\left(j\right)}(\Theta_{m}(p))\right\|}_{2}^{2}$$

and CV_ϵ_(*p*, *m*) is the mean square value of the average response error on *S*_*m*_:

(16) $${\text{CV}}_{\epsilon }(p,\;m)=\frac{1}{N}\sum_{n=1}^{N}{\left\|\epsilon_{n}(\Theta_{m}(p))\right\|}_{2}^{2}.$$

Here *N* is the assumed duration of the average response. The weights *w*_e_ and *w*_ϵ_ vary the emphasis between the one-step prediction error and average response error. In the analysis below, we set *w*_e_ and *w*_ϵ_ to the medians of CV_*e*_(*p*, *m*) and CV_ϵ_(*p*, *m*), respectively, for *m* = 1, …, *M* and all *p* considered. This approach places approximately equal emphasis on the two errors. The model order *p* is chosen as the *p* that minimizes CV(*p*) over the range of *p* evaluated.

Several practical issues require attention for computing the average response error. First, use of an average evoked response assumes the stimulus is nominally identical for each epoch. Second, care must be taken in computing the average response of the model Θ to the stimulus *x*^(*j*)^_*n*_ over epochs in *S*_*m*_ if the effects of preceding stimuli extend into *S*_*m*_. In such a case the brain is not “at rest” upon the arrival of the new stimulus in *S*_*m*_, but is still responding to the preceding stimulus. This situation occurs when the response time of the cortex is longer than the inter-stimulus interval. We mimic this aspect of the measured data when computing the average model response by presenting the entire train of stimuli to the model and averaging over the responses corresponding to epochs in *S*_*m*_.

### 3.4. Model quality assessment

A key assumption for the consistency of the OLS estimates is that the residuals **w**^(*j*)^_*n*_ be serially uncorrelated, that is, temporally white. Serial correlation in **w**^(*j*)^_*n*_ may be a sign of mis-specifying the model or incorrect selection of order (*p*, ℓ) (Hong, 1996; Duchesne and Roy, 2004). We use a consistency test developed in Duchesne and Roy (2004) to validate our models. Denote by Γ_**w**_(*r*) = E{**w**^(*j*)^_*n*_(**w**^(*j*)^_*n* − *r*_)^*T*^} the covariance at lag *r*, the hypotheses of interest are:

(17) $$\begin{array}{l} H_{0}:\Gamma_{\text{w}}(r)=0,\text{ for all }r\neq 0\text{ vs}.\\ H_{1}:\Gamma_{\text{w}}(r)\neq 0,\text{ for some }r\neq 0. \end{array}$$

Let the residual at time *n* in epoch *j* be ${\hat{\text{w}}}_{n}^{\left(j\right)}={\text{y}}_{n}^{\left(j\right)}-\hat{\Theta }{\text{ z}}_{n-1}^{\left(j\right)}$. Let *q*(·) be a window function of bounded support *L*, that is, *q*(*r*) > 0, for |r| ≤ *L* and *q*(*r*) = 0 for |*r*| > *L*. Suppose that the last epoch is of length longer than (*J* − 1)*L*, that is, *N*_*J*_ > (*J* − 1) *L*. The test statistic derived in Duchesne and Roy (2004) for testing *H*_0_ vs. *H*_1_ is

(18) $$T_{N_{c}}=\frac{N_{c}\sum_{r=1}^{L}q^{2}(r)\text{tr}[{\text{C}}_{\hat{\text{w}}}^{T}(r){\text{C}}_{\hat{\text{w}}}^{-1}(0){\text{C}}_{\hat{\text{w}}}(r){\text{C}}_{\hat{\text{w}}}^{-1}(0)]-d^{2}M_{N_{c}}(q)}{{\left[2d^{2}V_{N_{c}}(q)\right]}^{1/2}}$$

where *N*_*c*_ = ∑^*J*^_*j* = 1_ *N*_*j*_ − (*J* − 1)*L* and

(19) $${\text{C}}_{\hat{\text{w}}}(r)\;=\frac{1}{N_{c}}[\sum_{j=1}^{J-1}\sum_{n=r+1}^{N_{j}}{\hat{\text{w}}}_{n}^{\left(j\right)}{\left({\hat{\text{w}}}_{n+r}^{\left(j\right)}\right)}^{T}+\sum_{n=r+1+(J-1)(L-r)}^{N_{J}}{\hat{\text{w}}}_{n}^{\left(J\right)}{\left({\hat{\text{w}}}_{n+r}^{\left(J\right)}\right)}^{T}],$$

for *r* = 0, 1, …, *L*, are the estimated residual covariance matrices. The functionals *M*_*N*_*c*__(*q*) and *V*_*N*_*c*__(*q*) of *q*(·) and *N*_*c*_ are defined as (Duchesne and Roy, 2004):

(20) $$M_{N_{c}}(q)=\sum_{i\;=\;1}^{L-1}\left(1-\frac{i}{N_{c}}\right)q^{2}(i)$$

(21) $$V_{N_{c}}(q)=\sum_{i\;=\;1}^{L-2}\left(1-\frac{i}{N_{c}}\right)\left(1-\frac{\left(i+1\right)}{N_{c}}\right)q^{4}(i).$$

We use the Bartlett window defined as *q*(*j*) = 1 − |*j*/*L*|, *j* ≤ *L* and *q*(*j*) = 0, *j* > *L* with a window width *L* = ⌈ 3*N*^0.3^_*c*_ ⌉ as suggested in Duchesne and Roy (2004). For example, in our datasets the longest possible single epoch would have *N*_*c*_ = 3000 samples, which leads to the maximum value *L* = 34. Thus the test statistic Equation (18) is based on estimated residual covariance matrices at lags less than or equal to 34. Under the assumption that both **y**^(*j*)^_*n*_ and *x*^(*j*)^_*n*_ are stationary, the test statistic is one-sided and asymptotically standard normally distributed (see Duchesne and Roy, 2004, Theorem 1). It declares that the residuals are serially correlated if *T*_*N*_ > *z*_1 − α_ and are white otherwise, where *z*_1 − α_ is the value of the inverse cumulative distribution function of the standard normal distribution at 1−α and α is the significance level of the test.

## 4. Results

### 4.1. Model parameters

We have varying definitions and lengths of epochs throughout our data processing procedures. For detection of outlying epochs we choose all epochs to be of length *N*_*j*_ = 100 samples based on the time between subsequent current stimuli. In model estimation and assessment of residual whiteness, the epochs are defined as the maximum contiguous segments between the time segments removed by the outlier detection process. This minimizes the impact of the initial conditions **z**^(*j*)^_*n*_0__ required at the start of each epoch. Hence, *N*_*j*_ varies across epochs and conditions. In CV, the epoch lengths are set to be equal with *N*_*j*_ = 100. This, along with choosing the test sets *S*_*m*_ to contain approximately the same number of epochs, makes the test sets span roughly the same amount of time.

As shown in Table 1, the number of outlying epochs is generally larger in sleep than in wakefulness, most likely due to the presence of slow waves during sleep. The number of partitions of the available epochs used in the CV procedure for determining model order *p* and the corresponding model order is shown in Table 2. We did not consider model orders higher than *p* = 30. We also evaluated an unconnected model consisting of *d* univariate ARX models to assess the importance of the coupling or connectivity between channels. The univariate models were estimated by applying the procedure described above to each channel. With the exception of Subject B, stimulus location 1 (L1), the CV procedure picks a higher model order for the unconnected model and in many cases chooses the maximum order considered.

**Table 2:** **Model order parameters for wakefulness and sleep data sets**.

	**Wakefulness**			**Sleep**		
**Dataset**	**CV Part**.	**MVARX *p***	**ARX *p***	**CV Part**.	**MVARX *p***	**ARX *p***
Subject A, 1 mA	7	20	30	8	20	30
Subject A, 5 mA	7	26	30	11	26	26
Subject B, L1	10	30	28	8	30	24
Subject B, L2	10	18	22	7	22	30
Subject C	10	16	30	7	12	30

The whiteness test described in section 3.4 was applied to the residuals from all models using a significance level α = 0.1. Note that since exceeding the threshold implies the residuals are not white, use of a relatively large value for α leads to a more stringent test, that is, makes it easier to declare the residuals are not white. The MVARX models passed the whiteness test for every data set, while the unconnected models failed the test for every data set.

### 4.2. Evoked response model performance

**Figure 4:**
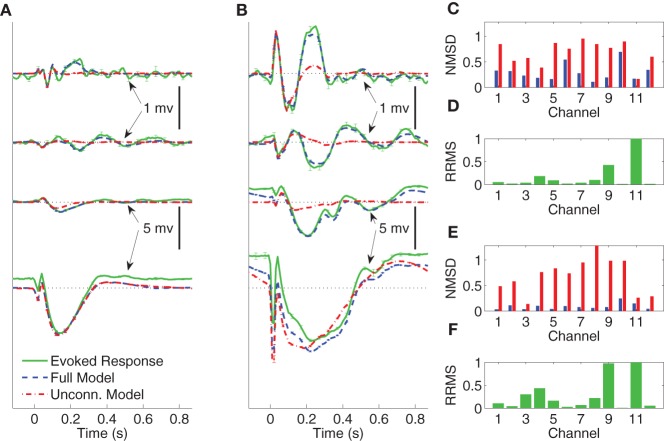
**Comparison between average CV evoked and average CV model responses of Subject A to two different stimulation strengths in wakefulness**. In panels **(A)** and **(B)** the black dotted lines indicate the origin while the error bars denote the standard error of the mean. **(A)** Average CV evoked and average CV model responses of channels 1, 7, 4, and 11 with 1 mA current stimulation. **(B)** Average CV evoked and average CV model responses of channels 1, 7, 4, and 11 with 5 mA current stimulation. **(C)** Normalized mean-squared difference in each channel for 1 mA stimulation. **(D)** Relative root mean-squared energy in each channel for 1 mA stimulation. **(E)** Normalized mean-squared difference in each channel for 5 mA stimulation. **(F)** Relative root mean-squared energy in each channel for 5 mA stimulation.

**Figure 5:**
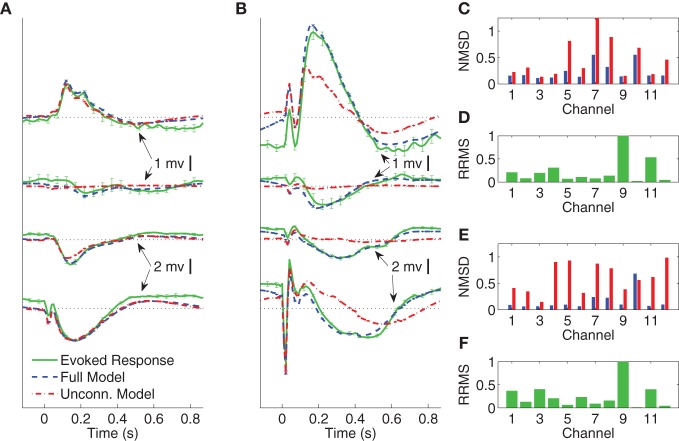
**Comparison between average CV evoked and average CV model responses of Subject A to two different stimulation strengths in sleep**. In panels **(A)** and **(B)** the black dotted lines indicate the origin while the error bars denote the standard error of the mean. **(A)** Average CV evoked and average CV model responses of channels 1, 7, 4, and 11 with 1 mA current stimulation. **(B)** Average CV evoked and average CV model responses of channels 1, 7, 4, and 11 with 5 mA current stimulation. **(C)** Normalized mean-squared difference in each channel for 1 mA stimulation. **(D)** Relative root mean-squared energy in each channel for 1 mA stimulation. **(E)** Normalized mean-squared difference in each channel for 5 mA stimulation. **(F)** Relative root mean-squared energy in each channel for 5 mA stimulation.

**Figure 6:**
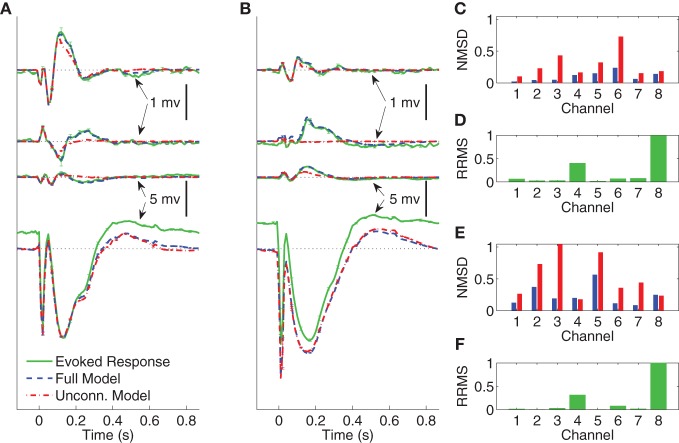
**Comparison between average CV evoked responses and average CV model responses of Subject B with two different stimulating locations in wakefulness**. In panels **(A)** and **(B)** the black dotted lines indicate the origin while the error bars denote the standard error of the mean. **(A)** Average CV evoked and average CV model responses of channels 1, 3, 6, and 8 when the stimulating channel is L1. **(B)** Average CV evoked and average CV model responses of channels 1, 3, 6, and 8 when the stimulating channel is L2. **(C)** Normalized mean-squared difference in each channel when the stimulating channel is L1. **(D)** Relative root mean-squared energy in each channel when the stimulating channel is L2. **(E)** Normalized mean-squared difference in each channel when the stimulating channel is L1. **(F)** Relative root mean-squared energy in each channel with the stimulating channel is L2.

In Figures 4–6 we compare the average evoked response and average model response for a subset of subjects and conditions. The average responses are generated following the CV approach described in section 3.3. Figures 4A,B show the average CV evoked responses ${\bar{\text{y}}}_{n}(S)=M^{-1}\sum_{m=1}^{M}{\bar{\text{y}}}_{n}(S_{m})$ and average CV model responses ${\hat{\bar{\text{y}}}}_{n}(\Theta ,\;S)=M^{-1}\sum_{m=1}^{M}{\hat{\bar{\text{y}}}}_{n}(\Theta_{m},\;S_{m})$ in channels 1, 4, 7, and 11 of Subject A in wakefulness for 1 and 5 mA stimulation, respectively. Here 0 s on the time axis corresponds to the stimulus onset. The averaging is first done within the testing block for each CV partition, then a second phase of averaging is done over the average responses of the test blocks for all CV partitions. The average CV model response of the MVARX model (blue dashed line) follows the dynamics of the average CV evoked response (green solid line) in each channel, for both stimulus amplitudes and a range of channel response levels. In contrast, the average CV model response of the unconnected model (red dashed dot line) only tracks the average CV evoked response in channels with the largest amplitudes, even though the univariate model is fit independently to each channel. In the figures, error bars indicating one standard error are displayed every five samples. Figures 4C–F summarize the model performance on a channel-by-channel basis. ${\bar{\text{y}}}_{i,\;n}(S)$ and ${\hat{\bar{\text{y}}}}_{i,\;n}(\Theta ,\;S)$ be the average CV evoked response and average CV model response at time *n* in the *i*-th channel. Figures 4C,E depict the normalized mean-squared difference (NMSD) between the average CV evoked and average CV model response for 1 and 5 mA stimulation, respectively, where the NMSD in channel *i* is defined as

(22) $$\text{NMSD}(i)=\frac{{\sum_{n=1}^{N}\left({\bar{y}}_{i,\;n}(S)-{\hat{\bar{y}}}_{i,\;n}\left(\Theta ,\;S\right)\right)}^{2}}{\sum_{n=1}^{N}{\bar{y}}_{i,\;n}^{2}(S)}.$$

Figures 4D,F depict the relative root mean-squared (RRMS) energy for 1 and 5 mA stimulations, respectively, for each channel. The RRMS for channel *i* is defined as the ratio of the root mean-squared energy in channel *i* to that of the channel with the largest root mean-squared energy. More precisely,

(23) $$\text{RRMS}(i)=\frac{\sqrt{\sum_{n=1}^{N}{\bar{y}}_{i,\;n}^{2}(S)}}{\underset{i^{\prime }=1,\;\ldots ,\;d}{\max}\sqrt{\sum_{n=1}^{N}{\bar{y}}_{i^{\prime },\;n}^{2}(S)}}.$$

The unconnected model only gives comparable NMSD to that of full model in channel 11, which has the largest energy. The difference between the MVARX model and the unconnected model in terms of per-channel NMSD is less significant for the 1 mA stimulation, than for the 5 mA stimulation.

Figures 5A,B depict the average CV evoked and average CV model responses for Subject A during NREM sleep with current stimulation of 1 and 5 mA, respectively. The four traces, from top to bottom, show the responses in channels 1, 7, 4, and 11, respectively. Panels (C) and (E) depict the NMSD, while (D) and (E) depict RRMS for 1 and 5 mA stimulation, respectively, as a function of channel.

The average CV evoked responses and the average CV model responses in wakefulness for Subject B, with two different stimulating sites L1 and L2, and both with current stimulus of 5 mA, are shown in panels (A) and (B) of Figure 6. The four traces, from top to bottom, depict the responses in channels 1, 3, 6, and 8, respectively. The difference between the two stimulating sites lies mainly in channels with smaller energy, i.e., channels 1, 3, and 6. Panels (C) and (E) depict NMSD in each channel when the stimulating channel is L1 and L2, respectively. Panels (D) and (F) show the RRMS in each channel.

Define the normalized mean-squared response difference (NMRD) over all channels as the ratio of the NMRD to the mean-squared average CV evoked response. That is,

(24) $$\text{NMRD}=\frac{\sum_{n=1}^{N}{\left\|{\bar{\text{y}}}_{n}(S)-{\hat{\bar{\text{y}}}}_{n}(\Theta ,\;S)\right\|}_{2}^{2}}{\sum_{n=1}^{N}{\left\|{\bar{\text{y}}}_{n}(S)\right\|}_{2}^{2}}.$$

**Figure 7:**
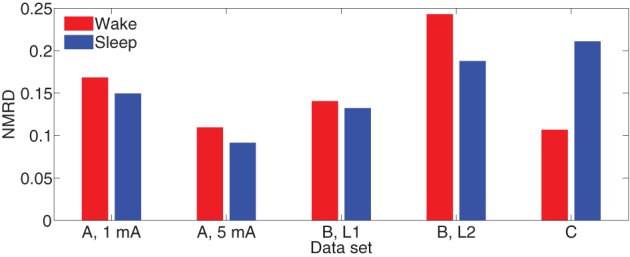
**Normalized mean-squared response difference [see Equation (24)] in each dataset**.

Figure 7 depicts NMRD of the MVARX models for all five data sets considered. Generally the MVARX models captures the dynamics in average evoked response reasonably well with NMRD no larger than 0.25.

### 4.3. One-step prediction model performance

**Figure 8:**
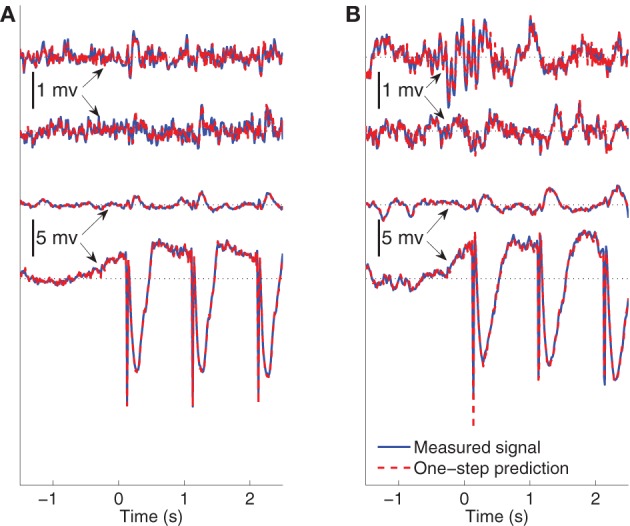
**Comparison between recorded signal and one-step prediction of Subject B when the stimulating site is L2**. 1.5 s pre-stimulus is shown followed by two-and-a-half epochs of evoked data. The black dotted lines in the panels indicate the origin. The model is estimated from data beginning with the fourth epoch. **(A)** Wake recorded and predicted signals in channels 1, 3, 6, and 8 ordered from top to bottom. **(B)** Non-REM sleep recorded and predicted signals in channels 1, 3, 6, and 8 ordered from top to bottom.

The ability of the model to predict the present recorded value of the data given past recordings reflects a different attribute than the modeling of the average evoked response. One-step prediction performance indicates the model's ability to follow spontaneous fluctuations in the data. Figure 8 compares the recording **y**^(*j*)^_*n*_ and one-step prediction ${\hat{\text{y}}}_{n}^{\left(j\right)}(\Theta )$ of the signals recorded from Subject B for 1.5 s of pre-stimulus data followed by two and a half epochs of evoked data, when the stimulating site is L2. The models used to perform prediction in Figure 8 are trained from data excluding the data plotted. Panels (A) and (B) shows the signals in wakefulness and sleep, respectively. Similar results are obtained for the other epochs, subjects, and conditions. The traces show the signals in channels 1, 3, 6, and 8, respectively. These results indicate that the MVARX model performs accurate one-step prediction in wakefulness and sleep and for both pre-stimulus and evoked data segments.

Define the normalized mean-squared one-step (NMSE) prediction error as the ratio of the mean-squared prediction error over the samples to the mean-squared energy. That is,

(25) $$\text{NMSE}=\frac{\frac{1}{J(N-n_{0})}{\sum_{j=1}^{J}\sum_{n=n_{0}+1}^{N}\left\|{\text{y}}_{n}^{\left(j\right)}-{\hat{\text{y}}}_{n}^{\left(j\right)}(\Theta )\right\|}_{2}^{2}}{\frac{1}{JN}{\sum_{j=1}^{J}\sum_{n=1}^{N}\left\|{\text{y}}_{n}^{\left(j\right)}\right\|}_{2}^{2}}.$$

**Figure 9:**
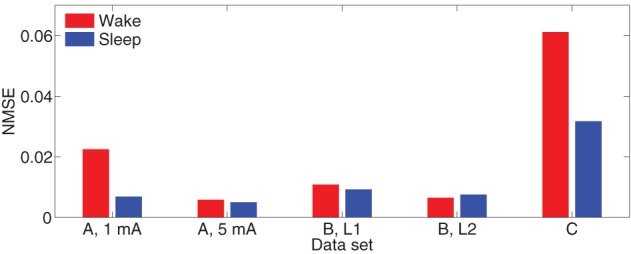
**Normalized mean-squared one-step prediction error [see Equation (25)] in each dataset**.

As a reference, the NMSE of the model Θ = **0** is approximately 1. The bar diagrams in Figure 9 show the NMSE of the MVARX models for all five datasets considered. Overall, our models give NMSE less than 0.06 for one-step prediction of the recordings and less than 0.02 in seven of the ten data sets studied.

### 4.4. B matrices

**Figure 10:**
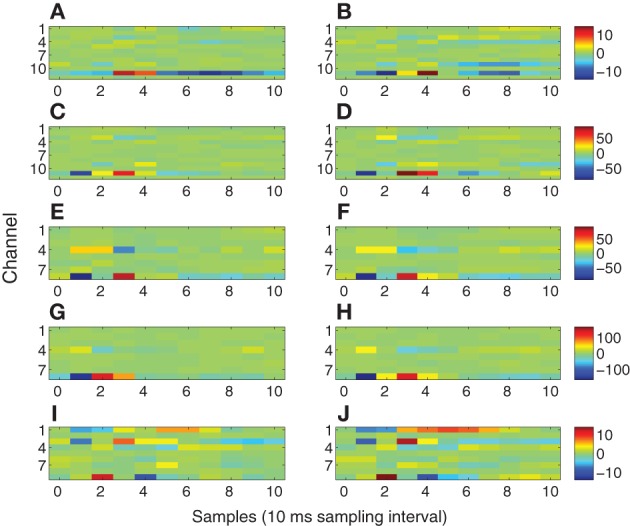
**Exogenous input filters **B** for each channel as a function of time**. The identical colormap is used for each row. **(A)** Subject A wake, 1 mA. **(B)** Subject A sleep, 1 mA. **(C)** Subject A wake, 5 mA. **(D)** Subject A sleep, 5 mA. **(E)** Subject B wake, stimulation site L1. **(F)** Subject B sleep, stimulation site L1. **(G)** Subject B wake, stimulation site L2. **(H)** Subject B sleep, stimulation site L2. **(I)** Subject C wake. **(J)** Subject C sleep.

Figure 10 depicts the exogenous input filters **b** matrices estimated for all 10 datasets as color plots. The *i*-th row of each matrix represents the FIR filter coefficients representing the path from the stimulus site to the *i*-th channel. Hence, rows with greater extremes of color have the strongest paths from the stimulus site.

## 5. Application to consciousness assessment

Numerous network characteristics can be obtained from an MVARX model. For example, graphs with partially directed coherence or conditional Granger causality as edges can be obtained by computing partially directed coherence or conditional Granger causality from the MVARX parameters. In this section we demonstrate the application of the model to assessment of consciousness by measuring the integrated information of the estimated MVARX model. The integrated information theory (Tononi, 2004, 2008, 2010) starts from two self-evident axioms about consciousness: every experience is one out of many and generates information because it differs in its own way from the large repertoire of alternative experiences; and every experience is one, that is, integrated, because it cannot be decomposed into independent parts. The theory formalizes these notions by postulating that a physical system generates information by reducing uncertainty about which previous states could have caused its present state, and that this information is integrated to the extent that it cannot be partitioned into the information generated by parts of the system taken independently. The theory predicts that integrated information in wakefulness is higher than that in sleep. Integrated information can be measured rigorously in models such as the MVARX model presented here. The integration of information is captured by **A** and **Q** in the MVARX model—**B** only indicates how stimulation enters the network. In this section we contrast integrated information in wakefulness and sleep using a variation on the procedure introduced in Barrett and Seth (2011) for obtaining a bipartition approximation to integrated information in MVAR systems. Our variation is based on use of “effective information” (Kullback–Leibler divergence) (Balduzzi and Tononi, 2008) in place of the difference in mutual information and ensures that integrated information is always positive (Cover and Thomas, 2006).

Suppose **y**_*n*_ describes a stable MVAR(*p*) process:

(26) $${\text{y}}_{n}=\sum_{i\;=\;1}^{p}{\text{A}}_{i}{\text{y}}_{n-i}+{\text{w}}_{n},$$

where **w**_*n*_ are i.i.d. zero-mean Gaussian noise vectors with covariance **Q**. Then the MVAR(*p*) process is wide sense stationary and ${\text{y}}_{n}\sim N(0,\;\Sigma (y))$ with Σ(**y**) = *E*{**y**_*n*_**y**^*T*^_*n*_}. Given that the state at time *n*, *y*_*n*_ = **y**, the conditional distribution of the state τ samples prior to sample *n*, **y**_*n* − τ_, follows

(27) $${\text{y}}_{n-\tau }|({\text{y}}_{n}=\underline{\text{y}})\sim N(\Gamma_{\tau }(\text{y})\Sigma {\left(\text{y}\right)}^{-1}\underline{\text{y}},\;\Sigma ({\text{y}}_{n-\tau }|{\text{y}}_{n}))\;$$

where Γ_τ_(**y**) = *E*{**y**_*n* − τ_**y**^*T*^_*n*_} and

(28) $$\Sigma ({\text{y}}_{n-\tau }|{\text{y}}_{n})=\Sigma (\text{y})-\Gamma_{\tau }(\text{y})\Sigma {\left(\text{y}\right)}^{-1}\Gamma_{\tau }{\left(\text{y}\right)}^{T}.$$

Given **A** and **Q**, the matrices Σ(**y**) and Γ_τ_(**y**) for τ = 1, …, ρ, with ρ ≥ *p* − 1, are computed as described in Barrett and Seth (2011).

Let the set of the channels be *S* = {1, 2, …, d}. A bipartiton ℬ = {*M*^1^, *M*^2^}, divides the channels into two mutually non-overlapping and non-empty sub-networks, *S* = *M*^1^∪*M*^2^. Denote two sub-systems **m**^1^_*n*_ and **m**^2^_*n*_ within which are the measurements in the channels corresponding to the elements in *M*^1^ and *M*^2^ at time *n*, respectively. Given Σ(**y**) and Γ_τ_(**y**), we have Σ(**m**^*i*^) = [Σ(y)]_*M*^*i*^, *M*^*i*^_ and Γ_τ_(**m**^*i*^) = [Γ_τ_(**y**)]_*M*^*i*^, *M*^*i*^_, for *i* = 1, 2. Hence, given the present state, the conditional distribution of the sub-system *i* at τ samples into the past is given by ${\text{m}}_{n-\tau }^{i}|({\text{m}}_{n}^{i}={\underline{\text{m}}}^{i})\sim N(\Gamma_{\tau }({\text{m}}^{i})\Sigma {\left({\text{m}}^{i}\right)}^{-1}{\underline{\text{m}}}^{i}$, Σ(**m**^*i*^_*n* − τ_|**m**^*i*^_*n*_)), for *i* = 1, 2, where Σ(**m**^*i*^_*n* − τ_|**m**^*i*^_*n*_) = Σ(**m**^*i*^) − Γ_τ_(**m**^*i*^)Σ(**m**^*i*^)^−1^Γ_τ_(**m**^*i*^)^*T*^.

Define the effective information for the system *y* over a lag of τ samples under partition ℬ as [see Barrett and Seth (2011), (0.32)]

(29) $$\varphi (y;\tau ,ℬ)=\frac{1}{2}[-\log_{2}\left(\det(\Sigma ({\text{y}}_{n-\tau }|{\text{y}}_{n}))\right)\;+\sum_{i\;=\;1}^{2}\log_{2}\left(\det(\Sigma ({\text{m}}_{n-\tau }^{i}|{\text{m}}_{n}^{i}))\right)]\text{bits}.$$

The effective information is the Kullback–Leibler divergence between a system consisting of two mutually independent sub-systems **m**^1^_*n*_ and **m**^2^_*n*_ and the system **y**_*n*_. The integrated information measured at a time difference of τ is defined as

(30) $$\phi (\text{y};\tau )=\varphi (\text{y};\tau ,{ℬ}^{\text{MIB}})$$

where the minimum information bipartion (MIB) is defined as

(31) $${ℬ}^{\text{MIB}}=\underset{ℬ}{\arg\min}\;\left(\frac{\varphi (y;\tau ,ℬ)}{K_{2}(ℬ)}\right)\;$$

with

(32) $$K_{2}(ℬ)=\min(H({\text{m}}_{n}^{1}),H({\text{m}}_{n}^{2}))$$

and the differential entropy of **m**^*i*^_*n*_, *H*(**m**^*i*^_*n*_) is given by

(33) $$H({\text{m}}_{n}^{i})=\frac{1}{2}\log_{2}\left({\left(2\pi e\right)}^{\left|M^{i}\right|}\det(\Sigma ({\text{m}}^{i}))\right).$$

**Figure 11:**
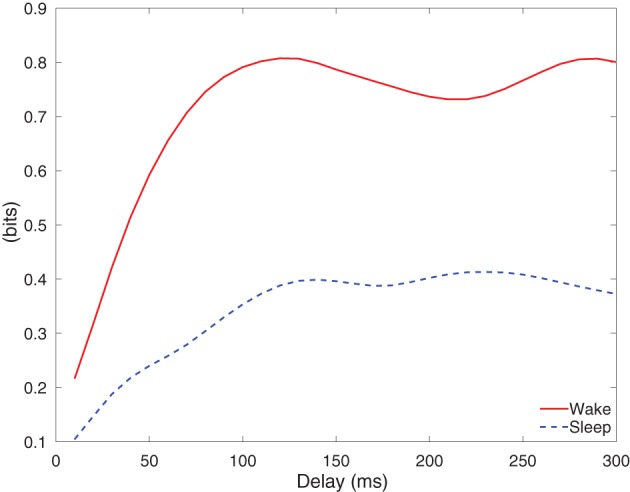
**Integrated information of Subject A, when the stimulation current is of 5 mA**.

**Figure 12:**
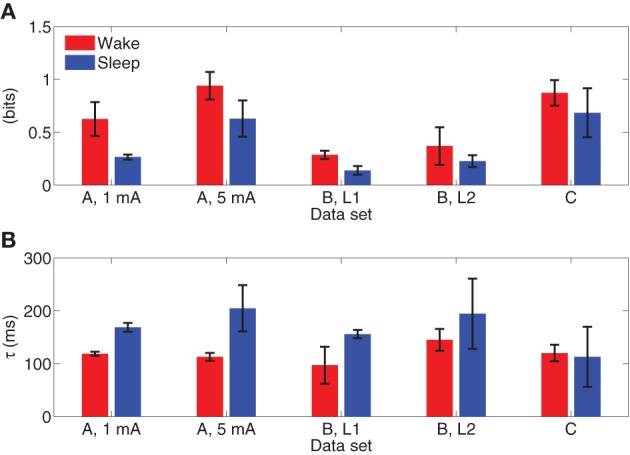
**(A)** Average maximum values of integrated information with error bars indicating one standard error. **(B)** Average lag at which maximum integrated information is achieved, with error bars indicating one standard error.

Figure 11 depicts the integrated information of Subject A for stimulus of 5 mA, as the time difference τ varies from 10 to 300 ms. The integrated information in wakefulness is higher than that in sleep. In both wakefulness and sleep, the integrated information increases until the time difference is approximately 100 ms and then remains approximately constant. We further used the CV procedures described in section 3.3 to study the difference between integrated information in wakefulness and sleep. Specifically, we estimated a model from the training set of each CV partition and compute integrated information for each CV partition. This provides *M* different estimates of integrated information for each data set, where *M* is the number of CV partitions. We compare the maximum values of the estimates of integrated information for each CV partition in wakefulness and sleep using the Wilcoxon rank sum test, which tests the null (*H*_0_) hypothesis that the measured maximum integrated information values in wakefulness and sleep for all CV partitions are samples from continuous distributions with equal medians, against *H*_1_ that they are not. The *p*-values of the rank sum test for each conditions are shown in Table 3. With the exception of Subject C, all of the cases have *p*-values below 0.05, and Subject C is only slightly above 0.05. Figure 12 depicts the average maximum value of integrated information and average time delay τ at which the maximum value is achieved, where the averaging is done across CV results, and error bars indicates one standard error.

**Table 3:** ***p*-values of the Wilcoxon rank sum test of whether integrated information in wakefulness and sleep are different**.

	**Subject A, 1 mA**	**Subject A, 5 mA**	**Subject B, L1**	**Subject B, L2**	**Subject C**
*p*-value	3.18e-4	0.0012	2.06e-4	0.0068	0.0553

## 6. Discussion

The results demonstrate the effectiveness of the MVARX model for intracerebral electrical stimulation data. Excellent agreement between measured and modeled evoked responses is found across channels, two stimulus amplitudes, vigilance states, stimulus sites, and subjects (Figures 4–7). One-step prediction is used to show that the MVARX model also accurately captures the spontaneous fluctuations in the measured signals (Figures 8 and 9). We contrast the MVARX models with a series of univariate ARX models, one for each channel, to illustrate the importance of accounting for the interaction between cortical signals (Figures 4–6). In some channels for some subjects/conditions the univariate ARX model describes the evoked response as well as the MVARX model. However, in general modeling interactions between cortical signals is necessary to capture the measured response. For example, in Figure 4B the univariate model fails to model the responses in channels 1, 4, and 7 beyond 200 ms after the stimulation.

The MVARX model explicitly represents both evoked and spontaneous (or background) brain activity using a deterministic input term to capture the effect of stimuli and a random input term to generate spontaneous activity. Stimuli generally give rise to a non-zero mean component in the response that varies with time, i.e., is non-stationary. Conventional approaches to MVAR modeling of cortical event-related potentials (e.g., Ding et al., 2000), subtract the ensemble mean of the data before processing to avoid the negative effects of the non-stationary mean on the MVAR model. However, subtraction of the ensemble mean significantly reduces the SNR of the data and is not necessary if the exogenous input is properly accounted for in the modeling procedure.

The effect of the stimulus on each recording channel is addressed by applying a separate filter in each channel to the stimulus signal. The filter coefficients are estimated jointly with the autoregressive model parameters from the measured evoked data. This approach accounts for the generally unknown and different characteristics of the transmission paths from the stimulation to each measurement site. The length of the filters [ℓ samples in Equation (5)] should be limited based on physiological expectations for the stimulus paradigm. Indeed, the autoregressive coefficients **A**_*i*_ and filters **b**_*i*_ are estimated simultaneously and the evoked response (**y**^(*j*)^_*n*, *e*_ in Equation (8)) is often much larger than the spontaneous component [**y**^(*j*)^_*n*, *s*_ in Equation (7)]. If ℓ is set equal to the duration of one epoch of **y**^(*j*)^_*n*, *e*_, then it is possible to perfectly model **y**^(*j*)^_*n*, *e*_ using only the **b**_*i*_ while setting the **A**_*i*_ = 0. We have shown that the MVARX models are capable of characterizing *y*_*n*, *s*_ by one-step prediction of data not used to estimate the model (see Figure 8). Moreover, the model describes the dynamics in **y**_*n*, *e*_, as was shown in Figures 4–6.

In order to define a practical value for ℓ we refer to previous electrophysiological studies on intracerebral evoked potentials (Matsumoto et al., 2004, 2007, 2012). In these studies Matsumoto and colleagues thoroughly discussed the possible generator mechanisms of intracerebral potentials evoked by direct electrical stimulation. In all of these studies it has been shown that the duration of the “purely evoked” response expires within 100 ms. Based on these results and our 100 Hz sampling frequency we set ℓ = 10. The 100 ms value is also consistent with our data. Indeed, the first 100 ms post-stimulus of the evoked waveforms exhibit quite different character than later portions. Typically the initial 100 ms of the measured response contain relatively sharp, high frequency waveforms, while later portions of the response have a smoother, lower frequency behavior. This suggests two regimes in the modeling process. The exogenous input filters account for the sharp initial response, as evident by the filter impulse responses shown in Figure 10. Channels having relatively large impulse response tend to rapidly transition from negative to positive maxima over one or two samples, consistent with the sharp features in the early portions of the evoked response. These sharp inputs to the channels are smoothed by the autoregressive component of the model to obtain the later portions of the response. The filter responses depicted in Figure 10 decay to relatively small values by the 10-th lag (100 ms) and generally contain most of their energy in the first through sixth lags, that is between 10 and 60 ms. This further supports the choice of ℓ = 10.

The energy transmission characteristics shown in Figure 10 are consistent with physiological expectations for modeling stimulation of fibers of passage. There is general consistency between wakefulness and sleep in all subjects (Figure 10, left column vs. right column) even though the evoked responses differ markedly (Figure 4 vs. Figure 5); channels with strong and weak responses are the same in wakefulness and sleep, and the shape of the responses in each channel are generally very similar. The subtle differences between wakefulness and sleep may be due to changes in neural excitability. Comparing 1 and 5 mA stimulation in Subject A (Figures 10A,B and C,D) reveals that channel 11 has the strongest response in both stimulation levels and the strength of the response increases roughly by a factor of 5, consistent with the factor of 5 change in the stimulation level. This is because we used the trigger signal to represent the exogenous input without adjusting its amplitude. However, the shape of the response in channel 11 differs slightly, with the 5 mA case having reduced latency by approximately 10 ms and a higher frequency response reflected by the sharper, shorter duration of the filter. This suggests that the higher stimulus level is associated with a faster response. The two stimulation sites L1 and L2 in Subject B (Figures 10E,F and G,H) both involve channels 8 and 4 as the strongest response, suggesting similar fibers of passage are excited at the two sites. However, the overall gain differs by a factor of 2 and the shape of the response in channel 8 and 4 differ, especially in wakefulness. Subject C (Figures 10I,J) exhibits multiple channels with strong linkage to the stimulus site.

Our MVARX approach assumes the dynamic interactions between evoked and spontaneous cortical signals follow the same model, that is, both evoked and spontaneous activity are described by one set of **A**_*i*_. The excellent one-step prediction performance in the pre-stimulus interval of Figure 8 combined with the high quality fitting of the evoked responses suggests this is a reasonable assumption, at least for these particular data sets. This approach also assumes that the measured signal is the sum of the evoked and spontaneous activity.

The windowed median filtering procedure successfully eliminated the volume conduction artifact while limiting changes to the measured signal to within ±20 ms of the stimulation. The outlier detection strategy only eliminates epochs that have significant deviation from the average evoked response. Both of these strategies significantly improve model fidelity to the measured data. Seven times as many outlier epochs were identified in sleep than in wakefulness, likely due to the presence of occasional slow waves during an epoch. However, in seven of the ten data sets we analyzed 28 or more of the 30 available epochs, which indicates our artifact detection procedure is not overly aggressive. Subject A had the most outlier epochs and in the worst case (5 mA, sleep) our procedure eliminated 8 of the possible 30 epochs. The CV strategy for choosing MVAR model order is effective, as demonstrated by the fidelity of the model evoked responses (Figures 4–7) and the ability of the models to accurately perform one-step prediction on pre-stimulus data (Figure 8). Outlier rejection helps the data meet the stationarity assumption of the MVARX model. While it is unlikely that the data are truly stationary, the accuracy with which the model describes the data and the whiteness of the residuals suggests that the stationarity assumption is reasonable.

As a proof of concept application, we used the MVARX model to assess changes in the level of information integration between wakefulness and deep sleep in human subjects. Using a simple, bipartition approximation we found that, as predicted by theoretical considerations (Tononi, 2004; Seth et al., 2008), integrated information is higher in wakefulness than sleep for each subject/condition, supporting the notion that integrated information reflects the capacity for consciousness. We note that the integrated information results presented here only apply to the recordings analyzed. Analysis of the dependence of integrated information on recording coverage is beyond the scope of this paper. Our findings indicate that the human cerebral cortex is better suited at information integration—being both functionally specialized and functionally integrated—when awake and conscious. In contrast, when consciousness fades in deep sleep, the parameters of the system change in such a way that information integration is diminished, in line with theoretical predictions (Tononi, 2004) and consistent with qualitative evidence obtained from experiments employing transcranial magnetic stimulation and high density EEG (Massimini et al., 2005). We also found that the lag at which the maximum level of information integration is attained is consistently longer in sleep than wakefulness. Maximum information integration in wakefulness occurred at lags of 30–110 ms, while those in sleep were from 70 to 140 ms longer, consistent with the increased low frequency activity of sleep.

### Conflict of interest statement

The authors declare that the research was conducted in the absence of any commercial or financial relationships that could be construed as a potential conflict of interest.

## References

[B1] Astolfi L. Cincotti F. Mattia D. De Vico Fallani F. Tocci A. Colosimo A. (2008). Tracking the time-varying cortical connectivity patterns by adaptive multivariate estimators. IEEE Trans Biomed. Eng. 55, 902–913 10.1109/TBME.2007.905419 18334381

[B2] Babiloni F. Cincotti F. Babiloni C. Carducci F. Mattia D. Astolfi L. (2005). Estimation of the cortical functional connectivity with the multimodal integration of high-resolution EEG and fMRI data by directed transfer function. Neuroimage 24, 118–131 10.1016/j.neuroimage.2004.09.036 15588603

[B3] Baccalá L. A. Sameshima K. (2001). Partial directed coherence: a new concept in neural structure determination. Biol. Cybern. 84, 463–474 11417058 10.1007/PL00007990

[B4] Balduzzi D. Tononi G. (2008). Integrated information in discrete dynamical systems: motivation and theoretical framework. PLoS Comput. Biol. 4:e1000091 10.1371/journal.pcbi.1000091 18551165 PMC2386970

[B5] Barnett L. Seth A. K. (2011). Behaviour of Granger causality under filtering: theoretical invariance and practical application. J. Neurosci. Methods 201, 404–419 10.1016/j.jneumeth.2011.08.010 21864571

[B6] Barrett A. B. Seth A. K. (2011). Practical measures of integrated information for time-series data. PLoS Comput. Biol. 7:e1001052 10.1371/journal.pcbi.1001052 21283779 PMC3024259

[B7] Bernasconi C. König P. (1999). On the directionality of cortical interactions studied by structural analysis of electrophysiological recordings. Biol. Cybern. 81, 199–210 10473845 10.1007/s004220050556

[B8] Bloomfield P. (2000). Fourier Analysis of Time Series: An Introduction. Wiley Series in Probability and Statistics, 2nd Edn New York, NY: Wiley-Interscience

[B9] Brovelli A. Ding M. Ledberg A. Chen Y. Nakamura R. Bressler S. L. (2004). Beta oscillations in a large-scale sensorimotor cortical network: directional influences revealed by Granger causality. Proc. Natl. Acad. Sci. U.S.A. 101, 9849–9854 10.1073/pnas.0308538101 15210971 PMC470781

[B10] Cheung B. L. P. Nowak R. D. Lee H. C. van Drongelen W. Van Veen B. D. (2012). Cross validation for selection of cortical interaction models from scalp EEG or MEG. IEEE Trans. Biomed. Eng. 59, 504–514 10.1109/TBME.2011.2174991 22084038 PMC3339867

[B11] Cossu M. Cardinale F. Castana L. Citterio A. Francione S. Tassi L. (2005). Stereoelectroencephalography in the presurgical evaluation of focal epilepsy: a retrospective analysis of 215 procedures. Neurosurgery 57, 706–718 16239883

[B12] Cover T. M. Thomas J. A. (2006). Elements of Information Theory. Wiley Series in Telecommunications and Signal Processing, 2nd Edn New York, NY: Wiley-Interscience

[B13] Dehaene S. Changeux J. P. Naccache L. Sackur J. Sergent C. (2006). Conscious, preconscious, and subliminal processing: a testable taxonomy. Trends Cogn. Sci. (Regul. Ed.) 10, 204–211 10.1016/j.tics.2006.03.007 16603406

[B14] Ding M. Bressler S. L. Yang W. Liang H. (2000). Short-window spectral analysis of cortical event-related potentials by adaptive multivariate autoregressive modeling: data preprocessing, model validation, and variability assessment. Biol. Cybern. 83, 35–45 10933236 10.1007/s004229900137

[B15] Ding M. Chen Y. Bressler S. L. (2006). Granger Causality: Basic Theory and Application to Neuroscience. Weinheim: Wiley-VCH Verlag GmbH & Co. KGaA 10.1016/j.jneumeth.2005.06.011

[B16] Duchesne P. Roy R. (2004). On consistent testing for serial correlation of unknown form in vector time series models. J. Multivar. Anal. 89, 148–180

[B17] Geweke J. F. (1984). Measures of conditional linear dependence and feedback between time series. J. Am. Stat. Assoc. 79, 907–915

[B18] Hong Y. (1996). Consistent testing for serial correlation of unknown form. Econometrica 64, 837–864

[B19] Kamiński M. J. Blinowska K. J. (1991). A new method of the description of the information flow in the brain structures. Biol. Cybern. 65, 203–210 1912013 10.1007/BF00198091

[B20] Korzeniewska A. Crainiceanu C. M. Kuś R. Franaszczuk P. J. Crone N. E. (2008). Dynamics of event-related causality in brain electrical activity. Hum. Brain Mapp. 29, 1170–1192 10.1002/hbm.20458 17712784 PMC6870676

[B21] Laureys S. (2005). The neural correlate of (un)awareness: lessons from the vegetative state. Trends Cogn. Sci. (Regul. Ed.) 9, 556–559 10.1016/j.tics.2005.10.010 16271507

[B22] Lütkepohl H. (2006). New Introduction to Multiple Time Series Analysis. Berlin: Springer

[B23] Malekpour S. Li Z. Cheung B. Castillo E. Papanicolaou L. Kramer A. (2012). Interhemispheric effective and functional cortical connectivity signatures of spina bifida are consistent with callosal anomaly. Brain Connect. 2, 142–154 10.1089/brain.2011.0058 22571349 PMC3621297

[B24] Massimini M. Ferrarelli F. Huber R. Esser S. K. Singh H. Tononi G. (2005). Breakdown of cortical effective connectivity during sleep. Science 309, 2228–2232 10.1126/science.1117256 16195466

[B25] Matsumoto R. Nair D. R. Ikeda A. Fumuro T. LaPresto E. Mikuni N. (2012). Parieto-frontal network in humans studied by cortico-cortical evoked potential. Hum. Brain Mapp. 33, 2856–2872 10.1002/hbm.21407 21928311 PMC6870075

[B26] Matsumoto R. Nair D. R. LaPresto E. Bingaman W. Shibasaki H. Lüders H. O. (2007). Functional connectivity in human cortical motor system: a cortico-cortical evoked potential study. Brain 130, 181–197 10.1093/brain/awl257 17046857

[B27] Matsumoto R. Nair D. R. LaPresto E. Najm I. Bingaman W. Shibasaki H. (2004). Functional connectivity in the human language system: a cortico-cortical evoked potential study. Brain 127, 2316–2330 10.1093/brain/awh246 15269116

[B28] McQuarrie A. D. R. Tsai C.-L. (1998). Regression and Time Series Model Selection. River Edge, NJ: World Scientific Pub Co Inc

[B29] Möller E. Schack B. Arnold M. Witte H. (2001). Instantaneous multivariate EEG coherence analysis by means of adaptive high-dimensional autoregressive models. J. Neurosci. Methods 105, 143–158 10.1016/S0165-0270(00)00350-2 11275271

[B30] Munari C. Hoffmann D. Francione S. Kahane P. Tassi L. Lo Russo G. (1994). Stereo-electroencephalography methodology: advantages and limits. Acta Neurol. Scand. Suppl. 152, 56–67 8209659 10.1111/j.1600-0404.1994.tb05188.x

[B31] Nobili L. De Gennaro L. Proserpio P. Moroni F. Sarasso S. Pigorini A. (2012). Local aspects of sleep: observations from intracerebral recordings in humans. Prog. Brain Res. 199, 219–232 10.1016/B978-0-444-59427-3.00013-7 22877668

[B32] Nobili L. Ferrara M. Moroni F. De Gennaro L. Russo G. L. Campus C. (2011). Dissociated wake-like and sleep-like electro-cortical activity during sleep. Neuroimage 58, 612–619 10.1016/j.neuroimage.2011.06.032 21718789

[B33] Penny K. I. (1996). Appropriate critical values when testing for a single multivariate outlier by using the mahalanobis distance. J. R. Stat. Soc. Ser. C (Appl. Stat.) 45, 73–81

[B34] Ranck J. B. (1975). Which elements are excited in electrical stimulation of mammalian central nervous system: a review. Brain Res. 98, 417–440 10.1016/0006-8993(75)90364-9 1102064

[B35] Rechtschaffen A. Kales A. (eds.). (1968). A Manual of Standardized Terminology, Techniques and Scoring System for Sleep Stages of Human Subjects. NIH Publication No. 204. Washington, DC: US Government Printing Office, National Institute of Health Publication

[B36] Seth A. K. Dienes Z. Cleeremans A. Overgaard M. Pessoa L. (2008). Measuring consciousness: relating behavioural and neurophysiological approaches. Trends Cogn. Sci. (Regul. Ed.) 12, 314–321 10.1016/j.tics.2008.04.008 18606562 PMC2767381

[B37] Tononi G. (2004). An information integration theory of consciousness. BMC Neurosci. 5:42 10.1186/1471-2202-5-42 15522121 PMC543470

[B38] Tononi G. (2008). Consciousness as integrated information: a provisional manifesto. Biol. Bull. 215, 216–242 19098144 10.2307/25470707

[B39] Tononi G. (2010). Information integration: its relevance to brain function and consciousness. Arch. Ital. Biol. 148, 299–322 21175016

[B40] Valentín A. Alarcón G. Honavar M. Garcia Seoane J. J. Selway R. P. Polkey C. E. (2005). Single pulse electrical stimulation for identification of structural abnormalities and prediction of seizure outcome after epilepsy surgery: a prospective study. Lancet Neurol. 4, 718–726 10.1016/S1474-4422(05)70200-3 16239178

[B41] Valentín A. Anderson M. Alarcón G. Garcia Seoane J. J. Selway R. Binnie C. D. (2002). Responses to single pulse electrical stimulation identify epileptogenesis in the human brain *in vivo*. Brain 125, 1709–1718 10.1093/brain/awf187 12135963

[B42] Winterhalder M. Schelter B. Hesse W. Schwab K. Leistritz L. Klan D. (2005). Comparison of linear signal processing techniques to infer directed interactions in multivariate neural systems. Signal Process. 85, 2137–2160

